# Effect of low dose honey on the apoptosis and inflammation gene expression in corneal limbal stem cells and keratocytes and its efficacy as an ophthalmic formulation in the treatment of dry eye: *in-vitro* and clinical study

**DOI:** 10.3389/fmed.2024.1359463

**Published:** 2024-05-20

**Authors:** Fatemeh Sanie-Jahromi, Mehdi Khaki, Mojtaba Heydari, Mohammad Hossein Nowroozzadeh, Amin Reza Akbarizadeh, Saeid Daneshamouz, Yaser NejatyJahromy, Maryam Nejabat, Ahmad Mahmoudi, Athar Zareei, Mahmood Nejabat

**Affiliations:** ^1^Poostchi Ophthalmology Research Center, Department of Ophthalmology, School of Medicine, Shiraz University of Medical Sciences, Shiraz, Iran; ^2^Department of Quality Control, Food and Drug, School of Pharmacy, Shiraz University of Medical Sciences, Shiraz, Iran; ^3^Department of Pharmaceutics, School of Pharmacy, Shiraz University of Medical Sciences, Shiraz, Iran; ^4^HIV/AIDS Research Center, Institute of Health, Shiraz University of Medical Sciences, Shiraz, Iran

**Keywords:** dry eye, honey, apoptosis, inflammation, corneal limbal stem cell, keratocytes

## Abstract

**Background:**

The use of honey as an eye treatment encounters challenges due to its high osmolarity, low pH, and difficulties in sterilization. This study addresses these issues by employing a low concentration of honey, focusing on both *in-vitro* experiments and clinical trials for treating dry eye disease in corneal cells.

**Methods:**

In the *in-vitro* experiment, we investigated the impact of a 1% honey-supplemented medium (HSM) on limbal stem cells (LSCs) and keratocytes using the 3-(4,5-dimethylthiazol-2-yl)-2,5-diphenyl-2H-tetrazolium bromide (MTT) assay and real-time polymerase chain reaction (PCR) for BCL-2, BAX, and IL-1β gene expression. Simultaneously, in the clinical trial, 80 participants were divided into two groups, receiving either a 1% w/v honey ophthalmic formulation or a placebo for 3 months. Study outcomes included subjective improvement in dry eye symptoms, tear break-up time (TBUT), and Schirmer’s test results.

**Results:**

MTT results indicated that 1% HSM did not compromise the survival of corneal cells and significantly reduced the expression of the IL-1β gene. Additionally, participants in the honey group demonstrated a higher rate of improvement in dry eye symptoms and a significant enhancement in TBUT values at the three-month follow-up. However, there was no significant difference between the study groups in terms of Schirmer’s test values. No adverse events were observed or reported.

**Conclusion:**

In conclusion, 1% honey exhibits anti-inflammatory and anti-infective properties, proving effective in ameliorating dry eye symptoms and enhancing tear film stability in patients with dry eye disease.

**Clinical Trial Registration**: https://irct.behdasht.gov.ir/trial/63800.

## 1 Introduction

Dry eye syndrome is a prevalent condition characterized by insufficient tear production or an imbalance in tear film composition, causing discomfort and visual disturbances (1). Its global prevalence ranges from 5 to 50%, varying based on geographical region and diagnostic criteria (2, 3). The condition significantly impacts the quality of life, leading to symptoms such as ocular discomfort, foreign material sensation, eye redness, blurred vision, and occasional pain (4). Existing treatments primarily focus on symptomatic relief and improving tear quality, including the use of artificial tear substitutes, lubricants, anti-inflammatory agents, and punctal occlusion (5). Despite these options, many patients continue to experience persistent symptoms, emphasizing the limitations of current treatments (6). Some individuals may also exhibit intolerance or inadequate response to conventional therapies, prompting the exploration of alternative approaches (7). Molecular and cellular events contribute to dry eye disease, with inflammation and corneal epithelial cell apoptosis identified as significant factors (8, 9). Anti-inflammatory treatments have shown utility in stimulating tear production and alleviating general dry eye symptoms (10).

Honey, a natural substance with recognized antimicrobial, anti-inflammatory, anti-cancer, and wound-healing properties, has been traditionally used for various therapeutic purposes, including eye-related conditions (11–17). While scientific evidence supporting honey’s ophthalmic use is limited, several studies have explored the efficacy of honey eye drops in managing dry eye, with promising results (18–21). However, varying honey concentrations (16.5 to 70%) in these studies raised concerns about increased osmolarity, acidity, and viscosity of eye drops, potentially causing irritation and discomfort (22). In this research, we addressed these challenges by using a diluted concentration of honey solution for both experimental (*in-vitro*) and clinical studies, focusing on corneal cells for managing dry eye disorder.

Our research aimed to evaluate the effects of a 1% w/v honey-supplemented medium (HSM) on the expression of apoptotic and inflammatory genes (BCL-2, BAX, and IL-1β) in corneal limbal stem cells (LSCs) and stromal keratocytes. Additionally, we assessed the anti-bacterial activity of HSM. Subsequently, we formulated an ophthalmic solution using 1% honey for the treatment of dry eye disease. Our focus on a lower dose aimed to mitigate potential osmolarity-related irritation and enhance overall treatment acceptability. Through our research, we aspire to contribute valuable insights into the therapeutic potential of a 1% honey ophthalmic formulation in managing dry eye syndrome, prioritizing patient comfort and tolerability.

## 2 Methods and materials

### 2.1 *In-vitro* experiment

#### 2.1.1 LSCs and keratocyte culture

Corneal tissue was obtained from a cadaver (a thirty-two years-old male) and used for corneal transplantation up to 48 h postmortem, the residual tissue after transplantation was transferred to the cell culture lab, under sterile conditions to be used for cell culture (23). Limbal biopsy (approximately 1 × 2 mm^2^) was extracted from the limbus and cultured in DMEM/F12 (Shell max, Iran) supplemented with 10% fetal bovine serum (FBS, Gibco, Germany) and 1% penicillin/streptomycin (Shell max, Iran). LSCs explants were cultured so that the epithelial layer was upward. After 15 min for the initial adhesion of tissue explants to the plate surface, the culture medium was gently added to the edge of the plate and gently tilted to spread throughout the culture dish. Approximately 7 days after the initial culture, LSCs showed growth from the bottom of the samples. Every 5 days, half of the culture medium was replaced with a fresh culture medium. When the cultures reached about 60–70% of confluence, the cells were characterized for the expression of LSCs markers including cytokeratin 19, vimentin (immunocytochemistry), CD44, P63, and ABCG2 (flow cytometry), and used for further analysis. In this study we intended to investigate the effect of 1% HSM on LSCs in their original cell architecture. As LSCs are susceptible to enzyme treatment and lose their cell–cell connections, all experiments were conducted using LSCs from primary cultures. This approach was specifically chosen to ensure the preservation of the cells’ intrinsic properties and to minimize any potential alterations in gene expression or functionality that might occur. Primary LSCs provide a more physiologically relevant model for assessing the effects of interventions.

Keratocyte cell culture was performed using the peripheral stroma of the same corneoscleral rime (a thirty-two years-old male) as described previously (24–26). Under sterile conditions, the corneal rim was relocated to the culture room. After rinsing the tissue with sterile phosphate buffer (PBS), the epithelial and endothelial layers were removed by knife scratching, and the rest of the tissue was cut into small segments, each roughly 1 mm in size. Stromal keratocyte explants were cultured in DMEM/F12 (Shell max, Iran) enriched with 10% FBS (Gibco, Germany) as well as 1% penicillin/streptomycin (Shell Max, Iran). Medium change and subculture were performed regularly every two to three days. Cells from passages 5–7 were checked for the expression of keratocytes markers (*LUM* and *KERA*) using polymerase chain reaction (PCR) and applied for further analysis.

#### 2.1.2 Chemical analysis of honey

Wax-free coriander honey from Koozeasal Co. (Product ID: hd-4, Esfahan, Iran), was analyzed and confirmed to be free from toxins or microorganisms.

##### 2.1.2.1 Hydroxy methyl furfural measurement

Hydroxy methyl furfural (HMF) was measured using White’s spectrophotometric method. Honey samples were mixed with water and Carrez solutions, filtered, and analyzed using a UV–visible spectrophotometer at 284 and 336 nm. The HMF value was calculated using the provided equation.

##### 2.1.2.2 Measurement of reducing sugars

The Lane-Eynon technique was used to quantify reducing sugars. Fehling’s solutions A and B were mixed with honey, and titration was performed with methylene blue as an indicator.

##### 2.1.2.3 Fructose/glucose ratio measurement

Fructose and glucose (F/G) were measured enzymatically. Fructose content was calculated by subtracting glucose content from total reducing sugars.

##### 2.1.2.4 Sucrose measurement

Sucrose content was determined by subtracting total reducing sugars from total sugar content, multiplied by a correction factor.

##### 2.1.2.5 Proline measurement

Proline content was measured by dissolving honey samples, adding formic acid and ninhydrin solution, heating, and reading the absorbance at 520 nm after the addition of propanol-water solution.

#### 2.1.3 The process of making of honey-supplemented culture medium

Honey (at concentration of 1% w/v) was dissolved in DMEM/F12 (Shell Max, Iran) at 37°C, then filtered through a 0.22 μm filter, and kept at 4°C until the time of the experiment. Before usage, the pH of HSM was determined and verified in terms of neutrality (pH ~7.2–7.4). Then, HSM-treated cells underwent relative gene expression analysis and cell viability assays, and their results were contrasted to those of controls (culture media without honey).

#### 2.1.4 MTT assay

Using an MTT test, the impact of HSM on LSCs and keratocyte proliferation was assessed. Cells were cultured in a 96-well microplate at a concentration of 10^4^ cells per well, and exposed to either 1% HSM or control media for 24 h at 37°C. Subsequently, 10 μL of 3-(4,5-dimethylthiazol-2-yl)-2,5-diphenyl-2H-tetrazolium bromide (MTT) (5 mg/mL PBS; GoldBio, United States) was added to each well and kept at 37°C for approximately 4 h. Crystals of formazan are created by live cells using MTT. After that, 100 μL acidic SDS [10% SDS (Parstous, Iran) in 0.01 M HCL] was added to each well and incubated at 37°C overnight. SDS helps dissolve cell membrane and formazan crystals and produces a purple solution, which optical density (OD) reflects the number of living cells. The OD of each well was then assessed at 545 nm wavelength using a microplate reader (BMG Labtech, Germany) and was used to evaluate keratocyte and LSCs survival in HSM vs. control groups.

#### 2.1.5 RNA extraction, cDNA synthesis, and real-time PCR

LSCs and keratocytes (10^6^ cells per 10 cm^2^ culture dish) were exposed to 1% HSM medium for 24 h at 37°C. We used the RNeasy kit (Parstous Co, Iran) for total RNA extraction and the Easy cDNA Synthesis Kit (Parstous, Iran) for cDNA synthesis. AlleleID software (v.7.5, Premier Biosoft International, Palo Alto, CA, United States) was utilized for primer design of *BAX*, *BCL-2*, *IL-1β*, and *ACTB* (house-keeping gene) (Table 1). The StepOneTM Real-Time PCR machine (Applied Biosystems) was used to run real-time PCR using RealQ Plus Master Mix Green (Ampliqon). PCR reactions were comprised of 7.5 μL master mix, 1.5 μL F and R primers, and 6 μL of DNA (total volume of 15 μL). Every PCR run had a single-step temperature profile with 40 cycles of 95°C hold phase for 15 min, 95°C denaturation phase for 10 s, and 61°C annealing and extension phase for 45 s. According to the 2^−ΔΔCt^ formulation, relative RNA expression was quantified.

**Table 1:** The primer sequences of the genes under study.

Gene	Sense primer	Anti-sense primer	Product size
*BCL-2*	CCCGCGACTCCTGATTCATT	CAGTCTACTTCCTCTGTGATGTTGT	167 bp
*BAX*	TTCTGACGGCAACTTCAACTGG	CACAGGGCCTTGAGCACC	78 bp
*IL-1β*	AGCAACAAGTGGTGTTCTCC	TGGGATCTACACTCTCCAGC	153 bp
*KERA*	CAACTGTCGCACAATCAAC	GAAGATGAGGTCCATAACTGAA	166 bp
*LUM*	CTGGCTGATAGTGGAATACC	TTGATCTTGGAGTAGGATAATGG	200 bp
*ACTB*	GCCTCGCCTTTGCCGAT	CATGCCGGAGCCGTTGT	98 bp

#### 2.1.6 Bacterial cultures and antimicrobial susceptibility test (disc diffusion)

To assess the antimicrobial activity of 1% HSM on ocular pathogens, an antimicrobial susceptibility test was performed. Cultures of nine common human ocular pathogenic strains were obtained from the local hospital of the study. Species included *Escherichia coli*, *Pseudomonas aeruginosa*, *Klebsiella*, *Staphylococcus aureus*, *Staphylococcus epidermidis*, *Streptococcus viridans*, *Staphylococcus haemolyticus*, *Enterococcus faecalis*, and *Acinetobacter*. Every sample was cultured, and if microbial growth materialized, differential cultures and assays were run to distinguish between various bacterial strains. Disc diffusion method was used for antibacterial activity tests. Mueller–Hinton agar, placed into either 100 mm or 150 mm Petri dishes, was the medium utilized in this experiment. The agar had a pH in the range of 7.2 to 7.4. The Mueller–Hinton agar culture medium (Merck, Germany) was streaked to create a bacterial lawn after being inoculated with a straight saline suspension of isolated colonies that had been corrected to 0.5 McFarland turbidity standard. The plates were dried and the HSM stock solution was diluted to concentration of 1%. A volume of 25 μL of each dilution was loaded into sterile, blank discs 6 mm in diameter (Padtanteb, Iran), and the disks were fully dried before placing on the plates. Then the disk was gently placed on top of the agar and was lightly pressed down with the pancetta. After 16–18 h of incubation at 35°C, the area of inhibited bacterial growth was measured. Antibiotic disks as positive control used for the tests included: chloramphenicol, gentamicin, ciprofloxacin, and ofloxacin. Distilled water-loaded discs were used as negative controls, also a vehicle medium (culture medium without honey) was used as control. The concentration of the solution would be at its peak right adjacent to the disc and would gradually diminish as the distance from the disk increase. No colonies would grow in the zone of inhibition if the drug was effective against bacteria at doses higher than critical concentration, that is, the region of the agar where the concentration of the antibacterial agent was greater than or equal to this minimum effective concentration. This-together with the rate of antibiotic diffusion-was utilized to determine how susceptible the bacteria were to that specific antibiotic.

### 2.2 Clinical study

#### 2.2.1 Ethics and patient recruitment

The study protocol was approved by the ethics committee of Shiraz University of Medical Sciences (IR.SUMS.MED.REC.1400.548) and registered with the Iranian Registry of Clinical Trials (IRCT20220117053744N1). Patients diagnosed with dry eye disease based on clinical signs and symptoms were recruited for the study. Inclusion criteria included a Schirmer test result of less than 5 mm in 5 min and tear break time of less than 10 s. Patients with blepharitis, meibomian gland disorder, a history of taking tetracycline or oral corticosteroids within the past 3 months, ocular surface disorders, previous eye surgery, or a history of allergies were excluded from the study.

#### 2.2.2 Blinding and allocation concealment

To minimize bias and ensure the integrity of the study, blinding procedures were implemented. Both the participants and the researchers involved in data collection and analysis were blinded to the treatment assignment. The honey and placebo eye drops were visually indistinguishable, as they had the same appearance and packaging. Only the pharmacist responsible for preparing the eye drops had access to the treatment allocation information. Allocation concealment was maintained to prevent selection bias. The treatment assignments were concealed from the researchers enrolling participants and assigning them to their respective groups. The allocation sequence was generated by a randomization procedure using computer-generated random numbers. The treatment assignments were then placed in opaque, sealed envelopes sequentially numbered. Each envelope was opened only at the time of participant enrollment, ensuring that the treatment allocation remained concealed until the participants were assigned to their respective groups.

#### 2.2.3 Treatment procedure

The treatment group received honey eye drops, administered three times a day for 3 weeks. Follow-up evaluations were conducted 6 weeks after completing the treatment. The control group received placebo formulation following the same time and duration protocol.

#### 2.2.4 Preparation of honey eye drops

Honey eye drops were prepared in a clean room according to Good Manufacturing Practice (GMP) protocols. The concentration of honey used was 1% (w/v) in artificial tear eye drops. The honey solution underwent sterilization through 0.45 μm and 0.22 μm filtration, followed by packaging and labeling. Benzalkonium chloride (BAK) (5 μg/mL), hydroxypropyl methyl cellulose (0.001 g/mL) and dextran 70 (0.001 g/mL) was used in both the honey and placebo eye drops.

#### 2.2.5 Study outcomes

The subjective improvement in symptoms of dry eye, tear break-up time and Schirmer’s test results were used as study outcome. The ocular surface disease index (OSDI) questionnaire was employed to assess the subjective improvement of dry eye symptoms. All patients were asked regarding any adverse events experienced during the study period.

### 2.3 Statistical analysis

Three copies of each *in-vitro* experiment were performed. Data from *in-vitro* experiments were given as mean ± standard error of mean (SEM) and examined using GraphPad Prism software (version 6; San Diego, CA, United States) and an unpaired *t*-test. The significance threshold was established at *p* < 0.05. Descriptive statistics, such as mean and percentage, were used to analyze the clinical data. Statistical tests, including Mann–Whitney, Chi-square, and ANOVA tests, were performed using SPSS version 21 software to assess the significance of the clinical findings.

## 3 Results

### 3.1 *In-vitro* observations

#### 3.1.1 Characterization of LSCs and keratocytes

##### 3.1.1.1 Limbal stem cells characterization

**Figure 1:**
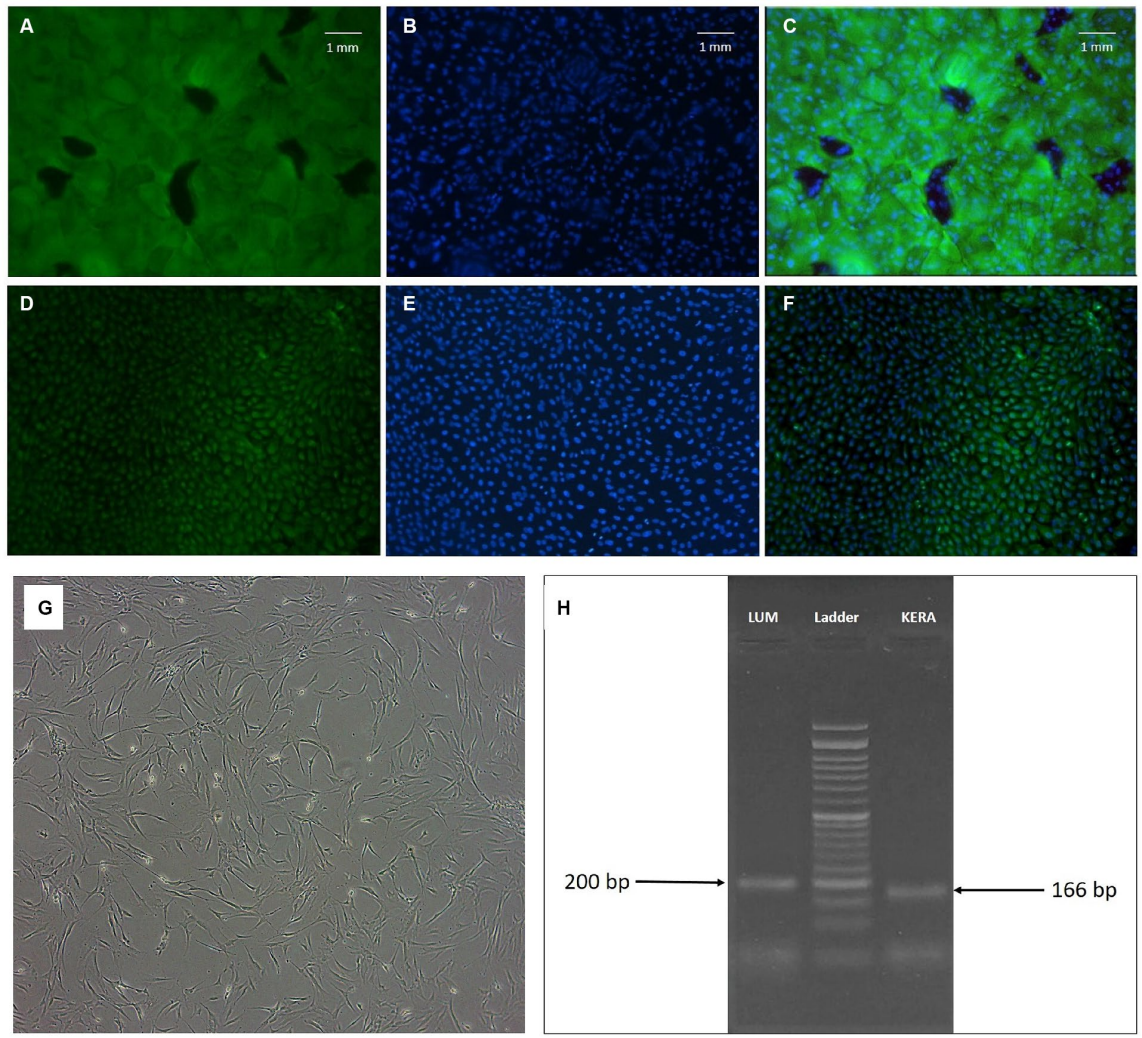
**(A–F)** Fluorescence microscopy of limbal epithelial stem cells. Positive cells for cytokeratin 19 **(A)** and vimentin **(D)** and their relevant DAPI field **(B,E)** are shown. **(C,F)** Fields represent the merged FITC and DAPI fields (magnification: ×100). **(G,H)** Keratocyte morphology and marker; **(G)**: phase contrast microscopic image of human corneal keratocytes (passage 5, magnification ×40). Note the fibroblastic morphology of the cells; **(H)**: agarose gel electrophoresis of *LUM* and *KERA* RT-PCR products of corneal keratocytes (24 and 26).

Our study acknowledged the complexity of limbal stem cell identification, adopting a multifaceted approach by incorporating both widely recognized (such as cytokeratin 19) and supplementary markers (such as vimentin). Cytokeratin 19 is a well-established marker of limbal epithelial cells. Vimentin is present in transitional limbal cells (27) and is a marker for EMT in limbal stem cells. Furthermore, by examining the cells’ biomolecular profiles in relation to their microenvironment, we utilized a panel of markers to identify and characterize LSCs. These included ABCG2, CD44, and P63, in accordance with the markers consensually associated with LSCs as highlighted in recent studies (28). Immunocytochemistry (ICC) results confirmed the identity of LSCs through the expression of cytokeratin 19 and vimentin markers in more than 90% of the cells (Figures 1A–F). The flow cytometry analysis revealed positive expression of CD44 (16.2%), P63 (75%), and ABCG2 (11.5%) as markers specific to LSCs. CD44 is a cell surface glycoprotein associated with cell adhesion, P63 is a transcription factor crucial for epithelial stem cell maintenance, and ABCG2 is an ATP-binding cassette transporter associated with stem cell properties. Together, these markers provided a comprehensive profile confirming the identity of limbal stem cells.

##### 3.1.1.2 Keratocytes characterization

Keratocytes displayed the characteristic spindle-shaped morphology indicative of these cells (Figure 1G). To further confirm their identity, the expression of *LUM* and *KERA* genes, recognized markers for keratocytes, was examined by PCR. The agarose gel electrophoresis showed the presence of expected bands at 200 bp for *LUM* and 166 bp for *KERA* (Figure 1H). Lumican (LUM) and keratocan (KERA) are proteoglycans associated with the extracellular matrix of corneal stroma and are considered specific markers for keratocytes. The detection of these bands in the PCR analysis provided additional validation of the identity of keratocytes in the study.

In summary, the use of cytokeratin 19, vimentin, CD44, P63, ABCG2, *LUM*, and *KERA* as markers, combined with various analytical techniques, ensured a thorough and reliable characterization of limbal stem cells and keratocytes in this investigation.

#### 3.1.2 Chemical analysis of coriander honey

Chemical analysis of coriander honey used in the ophthalmic formulation revealed notable characteristics, including a high percentage of reducing sugars (72.52% before and 73.56% after hydrolysis), low sucrose content (0.99 g/100 g), and balanced levels of fructose and glucose (36.3 g/100 g each, F/G ratio of 1.00). The presence of hydroxymethylfurfural (HMF) at 7.19 mg/kg, an indicator of honey quality, and a proline content of 734.7 mg/kg were also identified. These findings contribute valuable insights into the chemical composition of coriander honey, underscoring its significance as a key component in the ophthalmic formulation, potentially influencing the therapeutic efficacy and safety of the product. The summarized results are presented in Table 2.

**Table 2:** Chemical analysis of coriander honey used in the ophthalmic formulation.

Characteristics	Results	Test environment	
		T (°C)	H (%)
Reducing sugars before hydrolysis (%)	72.52	19.3	29
Reducing sugars after hydrolysis (%)	73.56	19.3	29
Sucrose (g/100 g)	0.99	19.3	29
Fructose (g/100 g)	36.3	19.3	29
Glucose (g/100 g)	36.22	19.3	29
F/G ratio	1.00	19.3	29
HMF (mg/kg)	7.19	19.3	29
Proline (mg/kg)	734.7	19.3	29

F/G, fructose/glucose, H, humidity; HMF, hydroxy methyl furfural, T, temperature.

#### 3.1.3 MTT assay

**Figure 2:**
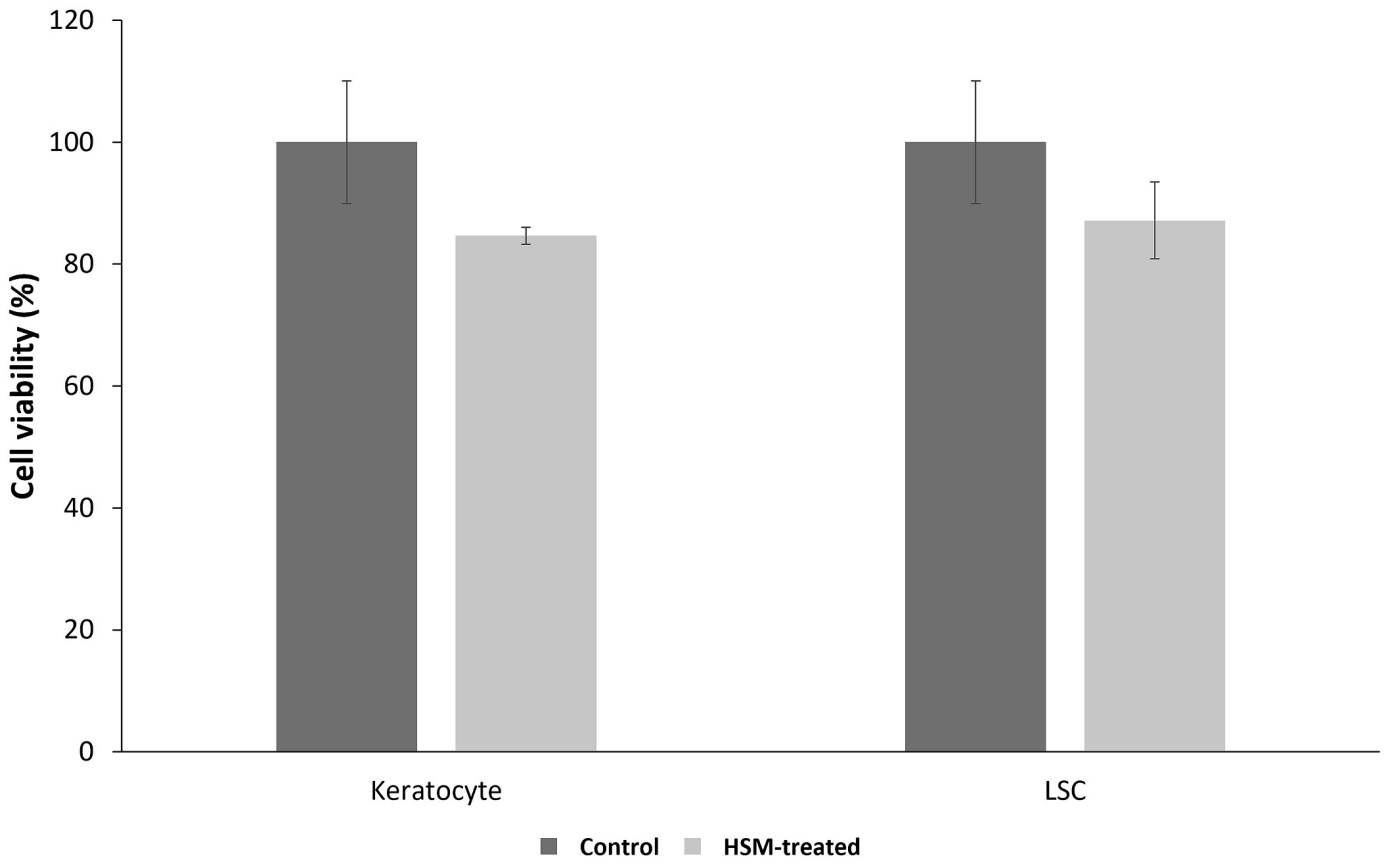
Cell viability of corneal LSCs and keratocytes after 24 h treatments with 1% HSM (treat, light bar) or the controls (dark bar) (*n* = 3, mean ± SEM). A *t*-test was used for statistical analysis.

The MTT assay results demonstrated that the 1% HSM did not adversely affect the survival of corneal LSCs and keratocytes, as evidenced by comparable viability to the control group. However, a non-significant reduction in cell proliferation was observed, with LSCs exhibiting 87.16 ± 10.03% viability compared to the control (100 ± 10.04%, *p* = 0.4167), and keratocytes showing 84.64 ± 1.37% viability compared to the control (100 ± 6.30%, *p* = 0.0758) (Figure 2).

#### 3.1.4 Real-time PCR

**Figure 3:**
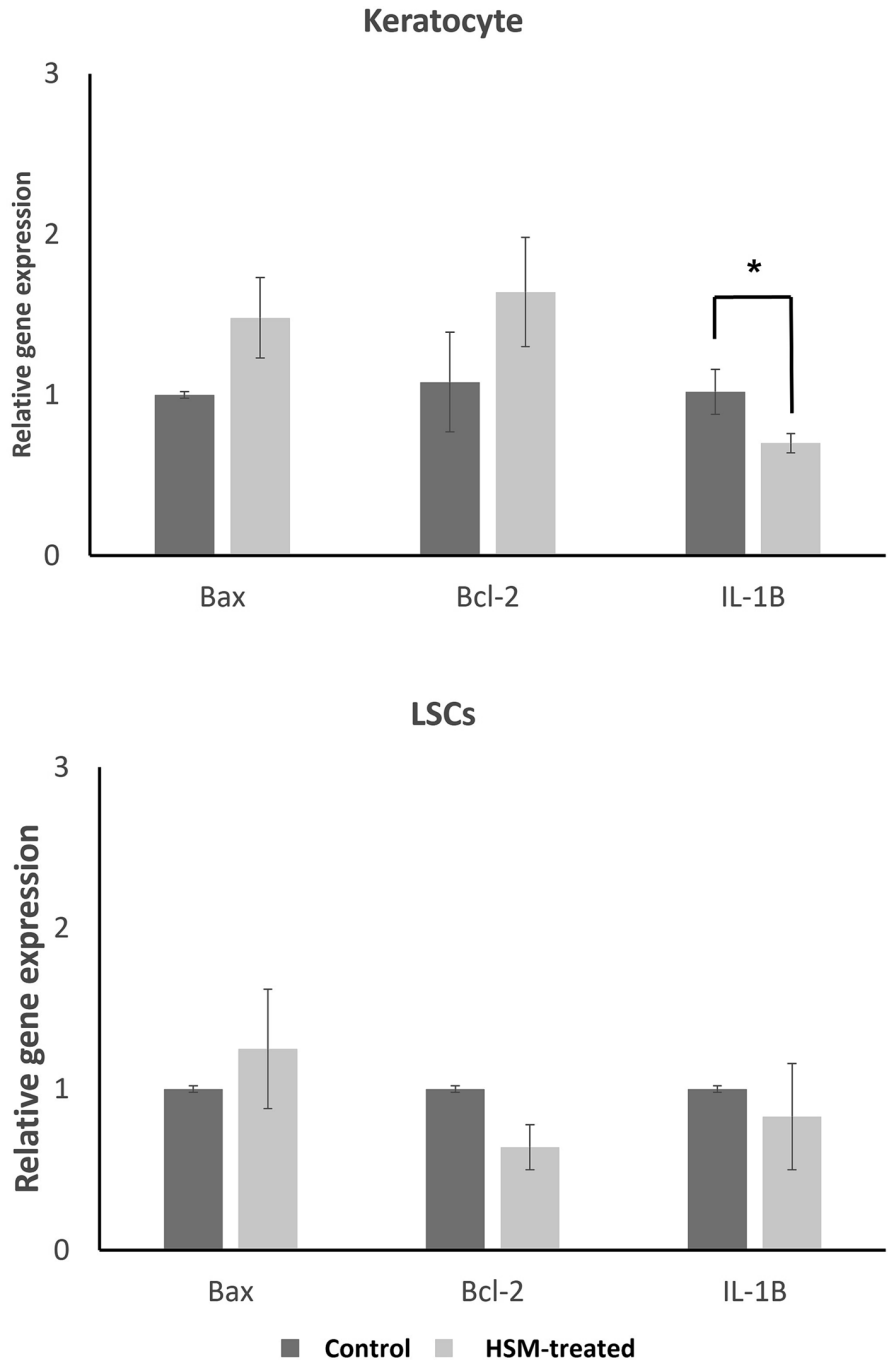
Relative gene expression of apoptotic genes (*BCL-2*, *BAX*) and inflammation marker (*IL-1β*) in LSCs and keratocytes treated by 1% HSM (treat, light bars) versus control (dark bars). The height of the bars indicates the mean value, and the error bars represent the SEM. Significant differences (*p* < 0.05) are highlighted with an asterisk (*). All pairs were compared using a student *t*-test.

The findings of our study indicate that the treatment of LSCs and keratocytes with 1% HSM did not result in a significant impact on the expression of BAX and BCL-2 genes, as illustrated in Figure 3. However, we observed a decreasing trend in the expression of the IL-1β gene in both LSCs and keratocytes treated with 1% HSM. Notably, this reduction reached statistical significance in keratocytes, indicating a potential anti-inflammatory effect of 1% HSM on these corneal cells. These results contribute to a better understanding of the molecular responses of LSCs and keratocytes to 1% HSM treatment, suggesting a specific influence on the expression of genes associated with inflammation in the keratocyte population.

#### 3.1.5 Bacterial test

The antibacterial activity of the 1% HSM solution was carefully evaluated by measuring the diameter of the zone of growth inhibition around the disks and comparing these measurements to those obtained with established antibiotics, including gentamicin, chloramphenicol, ciprofloxacin, and ofloxacin (Table 3). The length of the inhibitory zone is directly proportional to the inhibitory effect of the tested compound. Our analysis emphasizes that the notable antibacterial potential of 1% HSM was particularly evident against *Staphylococcus aureus* and *Staphylococcus epidermidis*, where it demonstrated comparable inhibitory effects in comparison to the reference antibiotics. Specifically, the zones of inhibition for these bacteria ranged from 10.00 mm to 15.44 mm, with *Staphylococcus epidermidis* showing the largest zone of inhibition at 15.44 mm. It is important to highlight that while 1% HSM showed promising results against these specific strains, it did not exhibit inhibitory effects against *Acinetobacter* and *Pseudomonas aeruginosa*. The vehicle medium (culture medium without honey) that was used as control showed growth for all the studied bacterial strains (see Table 3).

**Table 3:** The inhibition zone (mm) of 1% HSM in comparison to other antibiotics (in their clinically available concentration).

Bacteria	1% HSM	Gentamicin 0.3%	Chloramphenicol 0.5%	Ciprofloxacin 0.3%	Ofloxacin 0.3%
*Staphylococcus aureus*	13.5 (± 2.26)	16.33 (± 2.25)	17.67 (± 2.88)	21 (± 1.26)	14.33 (± 7.06)
*Staphylococcus epidermidis*	15.44 (± 4.36)	18.89 (± 6.01)	18.3 (± 6.30)	19.33 (± 6.61)	19.67 (± 6.12)
*Streptococcus viridans*	10 (± 0.89)	17.5 (± 0.71)	24.5 (± 2.12)	24 (± 2.83)	24 (± 2.83)
*Staphylococcus haemolyticus*	13.67 (± 5.69)	24.67 (± 3.21)	23.67 (± 3.79)	26.33 (± 0.57)	24.33 (± 2.89)
*Klebsiella*	11 (± 0.92)	21 (± 3.11)	17 (± 0.24)	26 (± 2.53)	26 (± 4.32)
*Escherichia coli*	10 (± 0.86)	22 (± 2.51)	27 (± 3.41)	27 (± 3.98)	26 (± 3.98)
*Enterococcus faecalis*	10 (± 1.20)	16 (± 1.11)	21 (± 0.98)	15 (± 0.99)	13 (± 0.98)
*Acinetobacter*	Growth	20 (± 0.89)	13 (± 0.76)	20 (± 1.45)	21 (± 1.34)
*Pseudomonas aeruginosa*	Growth	20.71 (± 1.38)	19.14 (± 2.79)	23.43 (± 2.51)	24.86 (± 2.04)

This outcome underscores the potential application of 1% HSM in targeting specific ocular pathogens and supports its consideration for inclusion in eye drop formulations, especially for infections known to be caused by susceptible bacterial strains.

### 3.2 Clinical outcomes

#### 3.2.1 Participants flow

**Figure 4:**
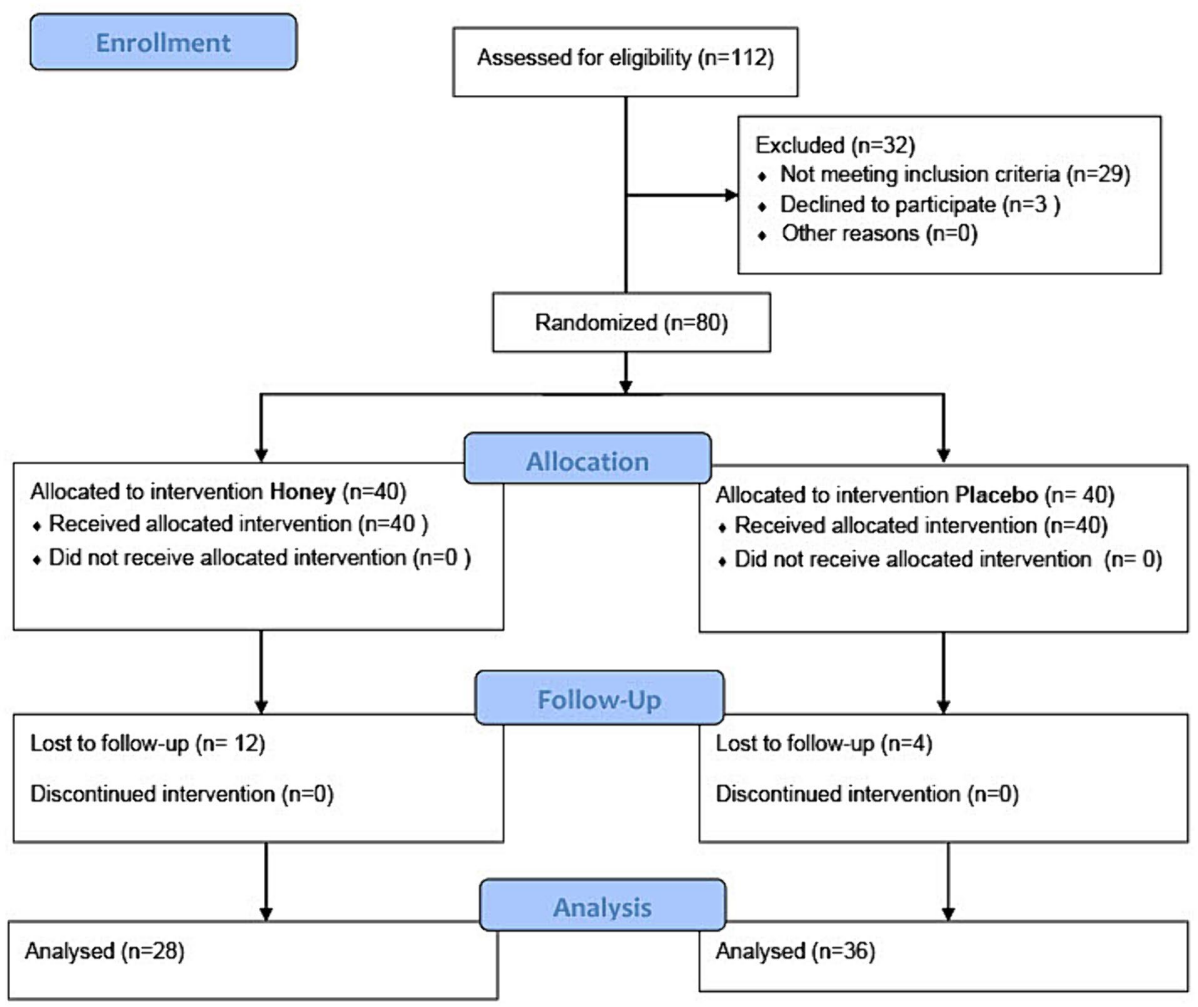
CONSORT flow diagram of the study showing the number of participants in each stage of study enrollment, allocation, follow-up, and analysis.

In the present study, out of the initially assessed 112 participants, 80 were randomized into either the honey intervention group (40 participants) or the placebo intervention group (40 participants). Four participants in the honey group and 12 in the placebo group were lost to follow-up. Consequently, 36 participants in the honey group and 28 participants in the placebo group were included in the final analysis. The participants’ flow is summarized in the CONSORT flow diagram (Figure 4).

##### 3.2.1.1 Basic characteristics

The baseline characteristics of participants in the honey and placebo groups were similar in terms of age and sex distribution. The mean age was 53.6 ± 11.8 years in the honey group and 53.1 ± 10.2 years in the placebo group. Sex distribution showed 47.5% females in the honey group and 43.3% females in the placebo group. Baseline measurements of Schirmer’s test and tear break-up time (TBUT) did not exhibit significant differences between the groups (Table 4).

**Table 4:** Baseline characteristics of the study participants.

Study groups	Honey	Placebo	*p*-value
Age, year	53.6 ± 11.8 (30 to 70)	53.1 ± 10.2 (30 to 70)	0.613^*^
Sex, female (%)	47.5%	43.3%	0.729^†^
Schirmer’s test, mm	4.7 ± 2.3 (1 to 9)	4.5 ± 2.2 (1 to 9)	0.837^*^
TBUT test, s	7.2 ± 2.2 (4 to 15)	7.5 ± 2.2 (5 to 13)	0.632^*^

^*^Calculated using Mann–Whitney *U* test. ^†^Calculated using Chi-squared test. Numeric data are presented as mean ± SD (range).

##### 3.2.1.2 Dry eye symptoms

The assessment of dry eye symptoms revealed a significantly higher percentage of participants experiencing improvement in the honey group (66.7%) compared to the placebo group (38.5%) (*p* = 0.029) (Table 5).

**Table 5:** Rate of improvement in dry eye symptoms in the two study groups.

Study groups	Honey, *n* (%)	Placebo, *n* (%)	*p*-value^*^
Improvement	22 (66.7%)	10 (38.5%)	0.029
No improvement	11 (33.3%)	16 (61.5%)	

^*^Calculated using Chi-squared test.

##### 3.2.1.3 Tear break-up time

TBUT values at baseline were comparable between the honey and placebo groups. At the three-month follow-up, the honey group demonstrated a significant improvement in TBUT (9.7 ± 2.2 s) compared to the placebo group (8.9 ± 3.3 s) (*p* = 0.008) (Table 6).

**Table 6:** Results of the tear break-up time test (in seconds) in the two study groups.

Study groups	Honey	Placebo	*p*-value
Baseline	7.2 ± 2.2 (4 to 15)^a,b^	7.5 ± 2.2 (5 to 13)	0.632^*^
Month 3	9.7 ± 2.2 (6 to 15)^b^	8.9 ± 3.3 (5 to 15)	0.326^*^
Improvement	2.97 ± 0.59	1.25 ± 0.51	0.008^*^
*p* value	< 0.001^†^	0.102^†^	

^*^Calculated using Mann–Whitney *U* test. ^†^Calculated using RM-ANOVA test. The pair-wise comparison was conducted by using Bonferroni correction. The same superscript letters show significant difference. Numeric data are presented as mean ± SD (range).

##### 3.2.1.4 Schirmer’s test

Baseline Schirmer’s test values were similar between groups, and both groups showed a significant increase at the three-month follow-up (*p* < 0.001). However, no significant difference was observed between the honey and placebo groups regarding the improvement in Schirmer’s test results (*p* = 0.554) (Table 7).

**Table 7:** Results of the Schirmer’s test (in seconds) in the two study groups.

Study groups	Honey	Placebo	*p*-value
Baseline	4.7 ± 2.3 (1 to 9)	4.5 ± 2.2 (1 to 9)	0.837^*^
Month 3	8.3 ± 5.7 (2 to 25)^b^	7.5 ± 4.8 (2 to 20)	0.554^*^
Improvement	3.72 ± 0.83	3.07 ± 0.81	0.548^*^
p value	< 0.001^†^	< 0.001^†^	

^*^Calculated using Mann–Whitney *U* test. ^†^Calculated using RM-ANOVA test. The pair-wise comparison was conducted by using Bonferroni correction. The same superscript letters show significant difference. Numeric data are presented as mean ± SD (range).

##### 3.2.1.5 Safety

No adverse events were observed or reported in patients treated with honey and placebo eye drops, indicating the safety of both interventions.

## 4 Discussion

Tear film instability, eye irritation, and vision impairment collectively characterize dry eye, a multifactorial ocular surface condition (29). From a molecular perspective, inflammation and apoptosis emerge as key signaling pathways in the pathogenesis of dry eye disease (30, 31). The historical use of honey in eye care, attributed to its anti-inflammatory and anti-apoptotic properties (32–34), positions it as a promising therapeutic candidate for managing dry eye.

Our *in-vitro* investigations affirmed the potential of a 1% honey concentration in supporting the proliferation of corneal cells. Specifically, this concentration demonstrated no significant impact on the expression levels of crucial genes associated with cell survival (BCL-2) and apoptosis (BAX) (35). The consistent BCL-2/BAX ratio in 1% honey-treated cells compared to controls implies the safety of this concentration for cell survival without promoting apoptosis in corneal cells. Moreover, a significant decrease in the expression of IL-1β, a pro-inflammatory cytokine linked to dry eye disease (36–38), underscores the anti-inflammatory potential of 1% honey (39–43).

The choice of IL-1β was made based on its well-documented involvement in the pathogenesis of dry eye disease and its established role as a key mediator of ocular surface inflammation. IL-1β is known to play a crucial role in initiating and perpetuating inflammatory responses, including the release of other inflammatory mediators and the disruption of ocular surface homeostasis. However, there are other significant inflammatory markers for dry eye disease, including MMP-9. Of course, exploring the expression profiles of additional inflammatory markers in future studies could provide valuable insights into the heterogeneity of dry eye disease phenotypes and facilitate the development of targeted therapeutic interventions tailored to individual patients’ inflammatory profiles.

In addition to the promising outcomes observed in the anti-inflammatory and apoptosis gene expression studies, our bacterial test results further underscore the multifaceted therapeutic potential of 1% honey. The observed antimicrobial activity of 1% honey against common ocular pathogens, including *Staphylococcus aureus* and *Staphylococcus epidermidis*, aligns with the growing body of evidence supporting honey’s broad-spectrum antibacterial properties (44–46). Notably, the inhibition zones measured for these bacteria suggest a potent bacteriostatic, if not bactericidal, effect of low-concentration honey, which is particularly relevant given the rising concern over antibiotic resistance in ocular infections (47–49).

Moreover, the significant inhibitory effect of 1% honey against certain strains compared to reference antibiotics suggests an exciting avenue for future research, and could pave the way for novel, natural antimicrobial agents in the fight against drug-resistant bacterial infections.

Of course, *in vivo* studies are essential to confirm the safety and efficacy of honey-based ophthalmic formulations, considering the complex microenvironment of the eye and potential variability in honey’s composition.

Translating these *in-vitro* insights into clinical outcomes, participants in the honey group exhibited a higher rate of improvement in dry eye symptoms and a significant enhancement in TBUT compared to the placebo group. Notably, Schirmer’s test values showed no significant difference between the study groups (18–21). This suggests that the honey formulation may primarily address tear film stability and ocular surface comfort, distinct from directly stimulating tear production.

To address challenges associated with high osmolarity and sterilization difficulties, we deliberately opted for a low concentration (1%) of honey. This choice allowed for adjustable osmolarity and facilitated easy sterilization through multiple filtrations. The selection of coriander honey further contributed to the study, bringing forth a unique composition with therapeutic properties encompassing antibacterial, antioxidant, anti-cancer, and anti-inflammatory effects (50–55).

Our findings resonate with previous studies demonstrating the efficacy of honey in managing dry eye symptoms (21, 56, 57). The improvement in TBUT, coupled with a reduction in subjective dry eye symptoms, aligns with research employing honey eye drops in various concentrations. The observed positive effects on meibomian gland dysfunction (MGD), a significant contributor to dry eye (58, 59), support the notion that honey contributes to ocular surface stabilization by reducing dryness, inflammation, and bacterial overgrowth.

In conclusion, our study supports the therapeutic potential of a 1% honey eye drop formulation in managing dry eye symptoms and improving tear film stability (25). The molecular effects observed *in vitro*, combined with positive clinical outcomes, suggest that honey, even at a low concentration, may influence corneal cells at the molecular level, contributing to its observed efficacy. Future research should delve into the precise mechanisms and potential synergistic effects of honey in dry eye management, advancing our understanding and refining treatment strategies.

It should be noted that the seasonal batch variation of honey is an important consideration in studies investigating its therapeutic properties, including our research on its efficacy in treating dry eye disease. While we made efforts to minimize the impact of this variability by sourcing honey from a consistent supplier and adhering to standardized processing methods, the inherent differences in honey batches may still exist. These differences could potentially affect the concentration and composition of bioactive compounds in the honey, consequently influencing its biological effects on corneal cells and clinical outcomes in dry eye patients. There is a certain need for future studies to explore strategies for standardizing honey preparations or incorporating methods to account for batch variability, such as rigorous quality control measures and detailed characterization of honey constituents.

## 5 Conclusion

**Figure 5:**
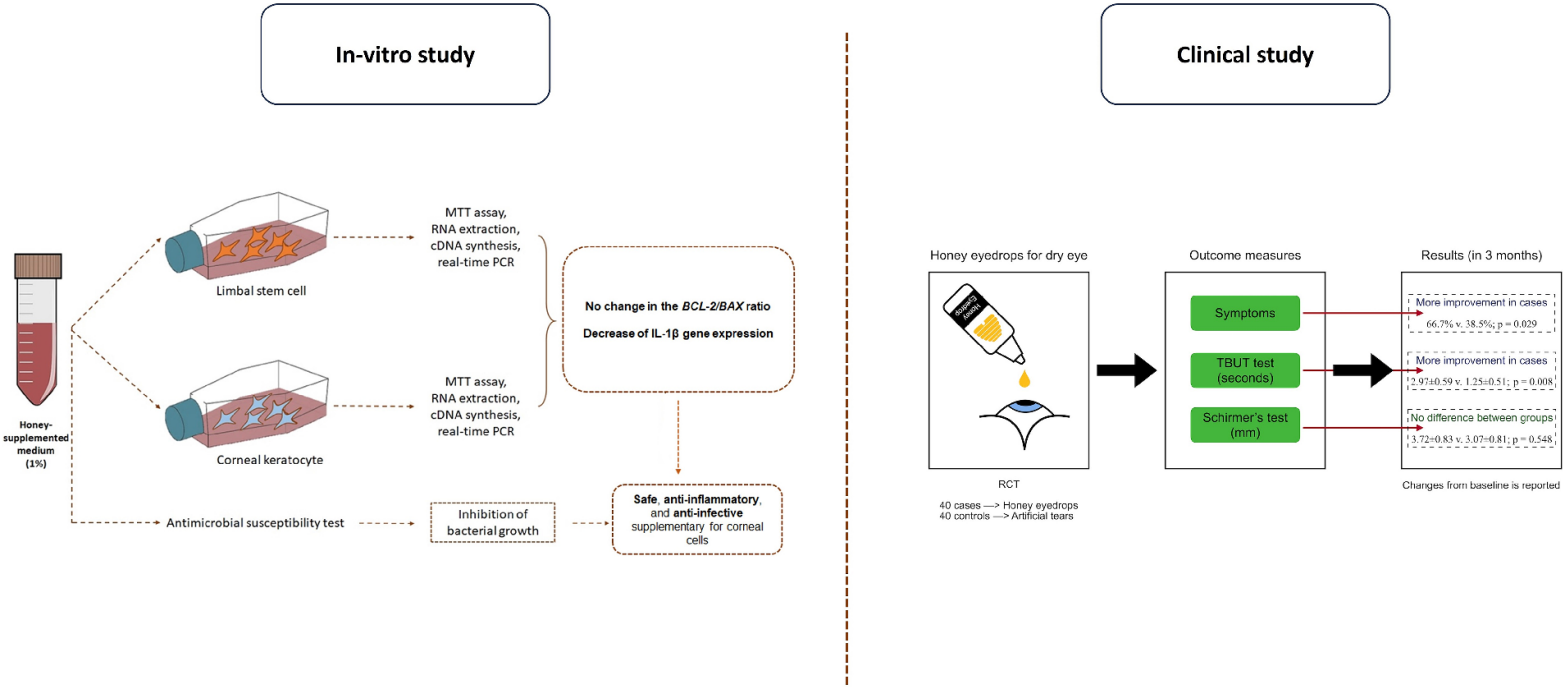
An overview of the study design and outcome.

In conclusion, our study highlights the suitability of a 1% honey solution as an effective anti-inflammatory and anti-bacterial supplement for corneal cells. The use of a 1% honey eye drop formulation demonstrates promising outcomes in ameliorating dry eye symptoms and enhancing TBUT as illustrated in Figure 5. Nonetheless, to validate and further understand these findings, additional research is essential. Future studies should focus on optimizing treatment protocols and delving into the potential mechanisms that underlie the therapeutic effects of honey in the management of dry eye. Continued investigation into honey-based treatments may contribute a valuable addition to the array of therapies available for individuals grappling with dry eye syndrome.

## Data availability statement

The raw data supporting the conclusions of this article will be made available by the authors, without undue reservation.

## Ethics statement

The studies involving humans were approved by Ethics Committee of Shiraz University of Medical Sciences. The studies were conducted in accordance with the local legislation and institutional requirements. Written informed consent for participation in this study was provided by the participants’ legal guardians/next of kin.

## Author contributions

FS-J: Conceptualization, Data curation, Investigation, Methodology, Project administration, Supervision, Validation, Writing – original draft, Writing – review & editing. MK: Data curation, Investigation, Methodology, Writing – original draft. MH: Formal analysis, Methodology, Writing – review & editing. MHN: Conceptualization, Formal analysis, Methodology, Software, Writing – original draft, Writing – review & editing. AA: Conceptualization, Data curation, Investigation, Methodology, Writing – original draft. SD: Conceptualization, Investigation, Methodology, Writing – original draft. YN: Writing – review & editing. MarN: Data curation, Investigation, Methodology, Writing – original draft. AM: Writing – original draft. AZ: Writing – original draft. MahN: Conceptualization, Funding acquisition, Investigation, Project administration, Supervision, Visualization, Writing – review & editing.
